# Family Determinants of Dental Fear and Anxiety Among Children Aged 6–8 Years in Jakarta, Indonesia: A Cross-Sectional Study

**DOI:** 10.3390/dj14070391

**Published:** 2026-06-24

**Authors:** Atik Ramadhani, Shafa R. Andini, Haslina Rani, Herry Novrinda, Febriana Setiawati, Vita Vianti, Armasastra Bahar

**Affiliations:** 1 Department of Dental Public Health and Preventive Dentistry, Faculty of Dentistry, Universitas Indonesia, Jalan Salemba Raya No. 4, Jakarta Pusat 10430, Indonesia; shafa.rizky@ui.ac.id (S.R.A.); herry_n@ui.ac.id (H.N.); febriana_s@ui.ac.id (F.S.); vita.vianti02@ui.ac.id (V.V.); armasastra.bahar@ui.ac.id (A.B.); 2 Department of Family Oral Health, Faculty of Dentistry, The National University of Malaysia (UKM), Kuala Lumpur 50300, Malaysia; hr@ukm.edu.my

**Keywords:** dental anxiety, children, parents, family determinants, Indonesia

## Abstract

**Background/Objectives:** Dental fear and anxiety (DFA) in children can negatively affect oral health behaviors and dental care utilization. Family-related factors, particularly parental anxiety, parenting styles, and socioeconomic characteristics, may be associated with DFA. This study aimed to investigate the association between family-related factors and DFA among children aged 6–8 years in Jakarta, Indonesia. **Methods:** A cross-sectional study was conducted among 294 child–parent pairs recruited from 10 primary schools using multistage cluster sampling. Children’s DFA was assessed using the Children’s Fear Survey Schedule–Dental Subscale (CFSS-DS), whereas parental dental anxiety was measured using the Modified Dental Anxiety Scale (MDAS). Sociodemographic and family-related characteristics, including parenting styles, were collected using self-administered questionnaires. Data were analyzed using chi-square tests and multivariable logistic regression. **Results:** Overall, 34.7% of the children were classified as having DFA. Maternal employment was significantly associated with children’s DFA, with children of formally employed mothers having higher odds of DFA (aOR = 2.01, 95% CI: 1.05–3.85; *p* = 0.034). Parental dental anxiety was associated with children’s DFA. Children whose fathers and mothers reported high levels of dental anxiety had 4.68-fold (95% CI: 1.64–13.33; *p* = 0.004) and 2.50-fold (95% CI: 1.10–5.74; *p* = 0.029) higher odds of experiencing DFA, respectively. Dental drilling and injections were the most frequently reported fear-provoking stimuli. The final regression model explained 13% of the variance in children’s DFA. **Conclusions:** Parental dental anxiety and maternal employment were significantly associated with DFA among children aged 6–8 years. Family-centered preventive strategies and early identification of at-risk children may help reduce DFA and promote positive dental experiences and oral health outcomes.

## 1. Introduction

Oral health is an important determinant of individual well-being. A Global Burden of Disease report in 2017 revealed that oral health affects approximately 3.5 billion individuals worldwide, with dental caries being the most prevalent condition. More than 2.3 billion individuals suffer from dental caries, and more than 530 million children are affected [1]. In Indonesia, the 2018 National Basic Health Survey (RISKESDAS) reported a prevalence of dental caries of 92.6% among children aged 5–9 years, with a mean decayed, missing, and filled teeth (DMFT) index of 0.7 [2]. Factors such as dental anxiety can contribute to the progression of dental caries, as some individuals avoid seeking dental care, leading to poor oral health [3].

Dental fear and anxiety (DFA), often arising in childhood, is a common psychological condition that may interfere with the delivery of dental care and contribute to poor oral health behaviors [4]. It ranks fifth among the most common conditions causing anxiety [5]. A systematic review revealed that the global prevalence of high levels of DFA is 15.3% [6]. A previous study revealed that the pooled prevalence rates of dental anxiety were 36.5% among preschoolers, 25.8% among schoolchildren, and 13.3% among adolescents [7]. In Jakarta, the reported prevalence rates of moderate to high levels of dental anxiety and fear among adults and elderly people were 16.3% and 36.1%, respectively [8]. Several factors have been associated with DFA, including painful dental experience, a dentist’s action, heightened sensitivity in the child, and inadequate psychological preparedness for dental care [9].

Dental anxiety appears during childhood as a result of vicarious learning. Environmental factors, such as family dynamics, play crucial roles in the development of DFA [10,11]. A previous study reported that factors such as treatment setting, child age, and parental occupation were significantly associated with DFA among children aged 8–12 years [12]. Other contributing factors included the dental anxiety of parents, the child’s birth order, family structure, and the presence of siblings [11,13]. Parenting style also plays a significant role in the development of DFA, as parenting provides an environmental context for children’s psychosocial development and influences their behavior [14]. Parenting styles have been a useful tool for studying the impact of parenting on several aspects of child development [15]. However, several studies have reported an association between parenting style and children’s DFA [16,17], whereas others have reported no such link [11,18].

Children aged 6–8 years are particularly vulnerable to oral health problems because they experience a substantially higher burden of untreated dental caries, with approximately twice the prevalence in primary dentition and five times the prevalence in permanent dentition, increasing their need for professional dental care [19]. A previous study revealed that the fear of dentists and dental treatment procedures predict untreated carious teeth in schoolchildren [20]. Understanding the factors associated with DFA in this age group is therefore important for improving children’s dental experiences and promoting timely utilization of dental services.

Jakarta is Indonesia’s largest metropolitan area and is characterized by substantial ethnic, cultural, and socioeconomic diversity. It has more than 10.6 million residents as of 2023, making it Indonesia’s most significant urban area [21]. This provides an important setting for investigating the factors associated with children’s DFA. However, to the best of our knowledge, few studies have reported DFA and associated factors in Jakarta [8]. Given the potential impact of DFA on oral health, treatment-seeking behavior, and quality of life, a better understanding of family influences may inform both clinical management strategies and preventive oral health policies. Therefore, this study aimed to explore the associations between family-related factors and the level of DFA among children aged 6–8 years in Jakarta.

## 2. Materials and Methods

### 2.1. Study Design

A cross-sectional study was conducted among children attending primary schools in Jakarta, Indonesia, between September and November 2023. The present study followed the recommendations of the Strengthening the Reporting of Observational Studies in Epidemiology (STROBE) statement [22].

### 2.2. Study Setting and Participants

The sample size was calculated via the formula [23] *n* = $\frac{Z\alpha^{2}P(1\;-\;P)}{d^{2}}$, where *n* represents the required sample size, *Z*α represents the standard normal value for a 95% confidence interval (1.96), *P* represents the estimated prevalence, and *d* represents the margin of error (0.05). The value of *P* was derived from a previous study reporting the prevalence of dental anxiety among children in Manado, Indonesia [24]. The calculations indicated that a minimum sample size of 250 participants was required, assuming a 5% margin of error and a 95% confidence level. To account for potential nonresponse and incomplete questionnaires, the required sample size was increased by 20%, resulting in a target sample of 300 child–parent pairs.

### 2.3. Sampling and Eligibility Criteria

A multistage cluster sampling technique proportional to population size was employed [25]. The sampling strategy was as follows: First, a list of public and private primary schools from the five administrative districts of Jakarta (south, east, west, central, and north Jakarta) was obtained. The data were obtained from the Ministry of Education, Culture, Research, and Technology in DKI Jakarta Province [26]. Public and private primary schools within each district were randomly selected using the onlineResearch Randomizer software (version 4.0) [27]. A total of 10 schools were included across the five districts, and they agreed to participate in the study.

Second, eligible students aged 6–8 years who were enrolled in grades 1 and 2 of the selected schools were randomly selected. The number of selected students was proportional to the required sample size and the student population of each district. This method resulted in the selection of 61 students from South Jakarta, 92 from East Jakarta, 30 from Central Jakarta, 68 from West Jakarta, and 49 from North Jakarta. The eligibility criteria for participants was that both the child and their parents were literate and able to complete the questionnaires independently. Children with systemic diseases or physical or psychological disabilities were excluded. Only randomly selected students whose parents provided written informed consent were included in the study.

Informed consent forms, participant information sheets, and questionnaires were distributed and collected through the schoolteachers. The questionnaires were completed independently by participants at home. To account for potential nonresponse and incomplete data, 401 questionnaires were initially distributed to ensure that the minimum required sample size was achieved. Of these, 344 were returned, yielding a response rate of 85.8%. After 50 incomplete questionnaires were excluded, data from 294 child–parent pairs were included in the final analysis.

### 2.4. Tools and Data Collection

The questionnaire consisted of four sections and required approximately 25 min to complete. Responses were provided anonymously, and participant confidentiality was maintained. In the first section, parents provided information on the child’s demographic background, including sex (male, female), age, type of school (public, private), family socioeconomic status (parental education levels and occupations), the presence of siblings, and the child’s birth order. Parental education was categorized as low (elementary or below and secondary school) or high (tertiary education or above). Parental occupations were categorized as formal or informal employment (unemployed or informal work). According to the BPS, Statistics Indonesia, workers are classified as informal if they are self-employed, supported by unpaid workers, engaged in unpaid family work, or work as free laborers in the agriculture or nonagricultural sectors. In contrast, workers who are employed with permanent contracts or paid wages are classified as formal sector workers [28].

The second section consisted of the Parenting Styles and Dimensions Questionnaire–Short Version (PSDQ), which was completed separately by fathers and mothers to assess authoritative, authoritarian, and permissive parenting styles [29]. In this study, a previously translated and validated PSDQ consisting of 32 items in Bahasa, Indonesia, was used [30]. The questionnaire included three subscales with good internal consistency (Cronbach’s alpha > 0.7): authoritative (Cronbach’s alpha 0.85), authoritarian (Cronbach’s alpha 0.81), and reasoning (Cronbach’s alpha 0.7). The responses to each item were made on a 5-point Likert scale ranging from “always” to “never.” The responses were scored on a 5-point Likert scale as follows: ‘never’ = 1, ‘sometimes’ = 2, ‘approximately half the time’ = 3, ‘often’ = 4, and ‘always’ = 5. The total score for each parenting style was calculated by summing the scores of the items in the corresponding parenting style. The parenting style with the highest mean score was considered the dominant parenting style for each parent.

In the third section, the modified dental anxiety scale (MDAS) was used to measure parents’ degree of dental anxiety. In this study, we used the Indonesian version of the MDAS, and its reliability and validity were documented [31]. The MDAS was selected because of its simplicity and good psychometric properties, including good validity (r = 0.706) and reliability (Cronbach’s α = 0.862). The questionnaire covers common dental situations, including visiting the dentist for treatment the following day, waiting in the dental clinic, performing tooth drilling, scaling and polishing procedures, and receiving a local anesthetic injection in the gums. The answers to each question in the MDAS questionnaire ranged from 1 (not anxious) to 5 (extremely anxious). The total score for the five items ranges from 5 to 25, with a higher score indicating a higher anxiety level. Dental anxiety was categorized as “low” (score range 5–14) or “high” (score ≥ 15) [32].

The fourth section consisted of the Children’s Fear Survey Schedule-Dental Subscale (CFFS-DS), which is used to measure children’s dental fear and anxiety (DFA) [33]. The Indonesian version of the CFSS-DS was used in this study, and the children completed it by themselves [34]. The CFSS-DS consists of 15 items, and for each item, the response options range from 1 to 5, from “not afraid at all” to “very afraid”. The Indonesian version demonstrated excellent internal consistency (Cronbach’s α = 0.966). The total score ranges from 15 to 75. A total score of the CFSS-DS below 32 is considered not fearful, and a score of ≥32 is defined as fearful [35].

### 2.5. Strategies to Address Potential Biases

Several strategies have been implemented to minimize potential bias. Participant anonymity was ensured to reduce social desirability bias, and validated, reliable questionnaires were used to minimize ambiguity and unclear items. To reduce selection bias, schools were selected using a random sampling procedure, clear eligibility criteria were applied, and efforts were made to achieve a high response rate among eligible participants. Given that the data were self-reported, the possibility of recall bias and response bias cannot be completely excluded.

### 2.6. Statistical Analysis

Data processing and analysis were conducted via IBM SPSS Statistics software version 26.0 for Windows (IBM, New York, NY, USA). Data processing began with data entry and coding, followed by univariate, bivariate, and multivariable logistic regression analyses. Descriptive statistics were used to summarize participants’ characteristics, whereas chi-square (χ^2^) tests were performed to examine associations between independent variables and children’s DFA. Variables considered theoretically relevant on the basis of previous literature and those demonstrating statistically significant associations in bivariate analyses were entered into the multivariable logistic regression model, which is consistent with a purposeful selection strategy [36]. The final model was developed using backward elimination, and the results are presented as adjusted odds ratios (aORs) with corresponding 95% confidence intervals (CIs). The fit of the model was assessed using the Hosmer–Lemeshow goodness-of-fit test, with a *p*-value > 0.05 indicating acceptable fit. Statistical significance was set at a *p*-value < 0.05.

### 2.7. Ethics

This study was approved by the Dental Research Ethics Committee, Faculty of Dentistry, Universitas Indonesia (Protocol No. 010730923) and was conducted in accordance with the principles of the Declaration of Helsinki. Written informed consent was obtained from all parents or legal guardians prior to participation.

## 3. Results

### 3.1. Participant Characteristics and Family-Related Factors Associated with Children’s Fear and Anxiety

The majority of the children were aged 7 years (47.3%), with a nearly equal distribution of males (52%) and females (48%). Most participants attended public schools (68.4%). A substantial proportion of fathers (68.7%) and mothers (72.4%) had low educational attainment. More than half of the fathers (51.7%) were formally employed, whereas the majority of mothers (81.3%) were engaged in informal work. The majority of the children had siblings (91.5%), and more than half were first-born (57.8%).

Both mothers and fathers predominantly exhibited an authoritative parenting style (79.3% and 97.3%, respectively), while authoritarian parenting was rarely reported (0.3% for both parents). Approximately one-third of the children (34.7%) were classified as having DFA. Parental dental anxiety levels were generally low, with 92.2% of fathers and 87.8% of mothers categorized as having low levels of anxiety on the basis of the MDAS.

Statistical analysis revealed a significant association between maternal occupation and children’s DFA (*p* = 0.020). Both maternal and paternal dental anxiety were significantly associated with children’s DFA (*p* = 0.001 for both). No statistically significant associations were observed between children’s DFA and other sociodemographic characteristics, family-related factors, or parenting styles (all *p* > 0.05) (Table 1).

Table 2 presents the results of the logistic regression analysis examining the factors associated with children’s DFA. Maternal employment was significantly associated with children’s DFA. Children whose mothers were formally employed had approximately twice the odds of experiencing DFA compared with those whose mothers were informally employed or unemployed (aOR = 2.01, 95% CI: 1.05–3.85; *p* = 0.034).

Parental dental anxiety was also significantly associated with children’s DFA. Children whose fathers reported high levels of dental anxiety were 4.68 times more likely to experience DFA than those whose fathers reported low levels of anxiety (aOR = 4.68; 95% CI: 1.64–13.33; *p* = 0.004). Similarly, children whose mothers had high levels of dental anxiety had 2.50 times greater odds of experiencing DFA than those whose mothers reported low levels (aOR = 2.50, 95% CI: 1.10–5.74; *p* = 0.029).

The overall model demonstrated acceptable fit, as indicated by the omnibus test of model coefficients (*p* = 0.001) and the Hosmer–Lemeshow goodness-of-fit test (χ^2^ = 1.045, *p* = 0.790). However, the overall explanatory power of the regression model was limited, with family-related factors accounting for only 13% of the variance in children’s DFA (Nagelkerke R^2^ = 0.13).

### 3.2. Children with Dental Fear and Anxiety (DFA)

Table 3 presents the distribution of children’s DFA across various dental-related stimuli. Most children were not afraid of visiting the dentist (51%), doctors (69.7%), or opening their mouth (87.1%). Similarly, common clinical interactions such as dental examinations and teeth cleaning were generally well tolerated, with 78.9% of the children reporting no fear of oral examinations and 68.4% reporting no fear of dental cleaning procedures.

In contrast, invasive procedures and unfamiliar experiences were associated with higher levels of fear. Dental drilling emerged as a prominent trigger, with 39.8% of the children reporting moderate fear and 23.2% reporting high to very high fear. Similarly, the sight and sound of the dental drill elicited moderate fear in 39.5% and 37.1% of the children, respectively. Dental injections were also a notable source of anxiety, with 17.7% of the children reporting moderate fear and 9.5% reporting high levels of fear. The median CFSS-DS score was 28 (range: 15–75), indicating generally low levels of DFA in the study population.

## 4. Discussion

The present study investigated family-related factors associated with dental fear and anxiety (DFA) among children aged 6–8 years in Jakarta, Indonesia. Although childhood DFA has been widely documented [4,9,10,11], evidence from Indonesia remains limited, particularly regarding the influence of family-related factors [8,24]. To our knowledge, this is among the first studies in Indonesia to comprehensively examine the associations between family characteristics, parental dental anxiety, parenting style, and DFA among children aged 6–8 years. Given the potential impact of DFA on oral health behaviors, treatment-seeking patterns, and long-term oral health outcomes, identifying family-related factors associated with childhood DFA may help inform both clinical management and preventive oral health strategies.

Approximately one-third of the children in this study exhibited DFA. This prevalence is consistent with findings from a previous systematic review reporting that approximately one-third of school-aged children experience DFA worldwide [7,37]. In contrast, a study conducted in Bandung, Indonesia, reported a substantially higher prevalence of 68% among younger children aged 3–6 years [38]. The discrepancy between these findings may be attributable to differences in age groups, study settings, measurement approaches, and sociocultural contexts.

The results revealed that among the dental-related stimuli assessed, tooth drilling emerged as the most commonly reported fear-provoking procedure. Previous studies have identified traumatic experiences, invasive clinical procedures, and acute dental pain as the predominant etiological factors in the development of dental anxiety [39,40]. Dental procedures such as drilling teeth and accompanying sounds have been reported as major triggers of dental fear [41]. These findings highlight the importance of considering multiple factors before initiating dental examination and treatment, as the development of dental fear during childhood may persist into adulthood and therefore requires early identification and management.

The study found no statistically significant associations between children’s or parents’ demographics and childhood DFA, except for the mother’s occupation. Children whose mothers were formally employed were more likely to exhibit DFA than those whose mothers were informally employed or unemployed. The role of mothers in children’s oral health behavior has been well documented [42,43]. Although previous studies have reported that maternal employment is associated with poor oral health outcomes among children [44], the mechanisms underlying this relationship remain unclear. One possible explanation is that employed mothers may have fewer opportunities to supervise their children’s oral health behaviors and accompany them to dental appointments [12,44]. Other factors, such as parenting stress and child temperament, may contribute to children’s DFA [45,46]. Furthermore, children whose mothers are less available during dental appointments may receive reduced parental reassurance, which could affect anxiety levels [47].

The present study demonstrated a significant association between both paternal and maternal DFA and children’s DFA. This relationship may be explained by the transmission of anxiety through parental modeling and verbal communication. Parents with dental anxiety may express fear in front of their children, which can reduce children’s self-efficacy in coping with dental-related pain and foster negative perceptions of dental care [48,49]. Such processes may increase vigilance during early dental encounters and heighten susceptibility to anxiety conditioning [49]. Moreover, parents’ negative experiences and implicit expressions of fear may contribute to the perception of dental care as threatening [50]. Anxious parents may also delay or avoid dental visits for their children, thereby limiting early exposure to nonthreatening dental experiences and increasing the likelihood that initial encounters involve invasive or painful procedures, reinforcing fear [49]. Therefore, familiarizing both parents and children with the role of dentists and common dental procedures may help reduce anxiety and promote greater utilization of preventive and treatment services.

In this study, there was no statistically significant difference between the parenting styles of fathers and mothers in terms of children’s dental anxiety. The results of this study are in line with those of previous studies [11,18]. Parenting style is thought to shape children’s behavior by providing an environmental framework for their psychosocial development, potentially reducing anxiety before they visit a dentist [17]. However, this association was limited to preschool children with no dental experience or dental phobia [51].

Although no statistically significant association was observed, these findings should be interpreted cautiously. The distribution of parenting styles in our sample was highly skewed, particularly among fathers, with authoritative parenting accounting for most responses. The small number of participants in the authoritarian and permissive categories may have reduced the statistical power to detect meaningful differences. Future studies involving more balanced distributions of parenting styles are needed to further examine the potential role of parenting practices in childhood DFA.

Our study revealed no statistically significant difference between the presence of siblings and child-birth order with DFA among children. Children with siblings can be directly or indirectly exposed to information about their sibling’s dental care or observe their sibling exhibiting anxious behavior during dental care. One possible explanation is that the influence of siblings on children’s perceptions of dental care may vary according to sibling relationships, personality characteristics, and previous dental experiences. Most studies have shown that compared with first-born children, middle-born children tend to have lower levels of anxiety and better behavioral outcomes in the dental setting [52]. In addition, less intimate relationships and warmth between siblings and parental favoritism toward older children may act as mediators between birth order and dental anxiety [52].

The final regression model explained 13% of the variance in children’s DFA. This modest explanatory power suggests that childhood DFA is a multifactorial condition influenced by a broader range of determinants beyond the family-related factors examined in this study. Previous research has identified several additional contributors to childhood DFA, including traumatic dental experiences, irregular dental attendance, untreated dental caries, and negative interactions with dental professionals [9,41,48]. Psychological characteristics such as child temperament, parenting stress, and coping ability may also play important roles [45,46]. Furthermore, parental oral health behaviors, attitudes toward dental care, and patterns of healthcare utilization may influence children’s perceptions of dental treatment [47,49]. Consequently, the present findings should be interpreted as highlighting the contribution of family-related factors within a broader biopsychosocial framework of childhood DFA.

Several limitations should be considered when interpreting the findings of this study. First, the use of a questionnaire-based survey may have introduced response-related biases, including social desirability bias, particularly for sensitive items related to parental education, occupation, and parenting practices. Second, the cross-sectional design precludes the establishment of temporal or causal relationships between family-related factors and children’s DFA. Third, although Jakarta is characterized by substantial ethnic and socioeconomic diversity, it represents a predominantly urban population and may not fully reflect the cultural, socioeconomic, and healthcare contexts of rural communities or other regions of Indonesia. Consequently, the findings may not be directly generalizable to all Indonesian populations, although they may provide valuable insights for settings with similar demographic characteristics. Furthermore, several potentially important determinants of childhood DFA, including previous traumatic dental experiences, a history of dental pain, caries severity, the frequency and nature of dental visits, child temperament, general anxiety traits, and parental oral health behavior toward dental care, were not assessed. The absence of these variables may have resulted in residual confounding and may partly explain the modest explanatory power of the regression model. Future longitudinal studies involving more geographically diverse populations and incorporating clinical, behavioral, and psychological factors are warranted to provide a more comprehensive understanding of the multifactorial determinants of childhood DFA. Nevertheless, the present findings underscore the importance of family-centered approaches, including psychological preparation and child-friendly dental care strategies, particularly for potentially anxiety-provoking procedures such as tooth drilling.

Children’s DFA is a multifactorial and complex condition influenced by both internal and external factors [53]. Community-based preventive programs that familiarize children and parents with dental procedures may help reduce DFA and improve dental service utilization. Parental education should emphasize the importance of routine dental care and encourage positive communication about dental experiences. Promoting positive parental role modeling, including avoiding negative comments about dental treatment in front of children, may further support the prevention of childhood DFA. In addition, evidence-based behavioral interventions, such as cognitive behavioral therapy (CBT), may be effective strategies for managing childhood dental anxiety [54].

## 5. Conclusions

Parental dental anxiety and maternal employment were significantly associated with dental fear and anxiety (DFA) among children aged 6–8 years. Children whose parents reported higher levels of dental anxiety were more likely to exhibit DFA, highlighting the potential influence of family-related factors on children’s perceptions of dental care. Although the regression model explained a modest proportion of the variance in childhood DFA, these findings suggest the multifactorial nature of childhood dental anxiety and suggest that family-related factors represent one important component of a broader biopsychosocial framework. Early identification and family-centered preventive strategies, including parental education and positive dental experiences, may help reduce dental anxiety and promote better oral health behaviors and the utilization of dental services. Longitudinal studies are needed to clarify the causal pathways linking family-related factors and childhood DFA.

## Figures and Tables

**Table 1:** Participant characteristics and bivariate association with children’s DFA (*n* = 294).

Variable	Total, *n* (%)	Children’s Dental Fear and Anxiety		*p*-Value
		Fearful (*n* = 102), *n* (%)	Not Fearful (*n* = 192), *n* (%)	
Children				
Sex				
Female	141 (48.0)	47 (46.1)	94 (49.0)	0.728
Male	153 (52.0)	55 (53.9)	98 (51.0)	
Age (years)				
6	29 (9.9)	12 (11.8)	17 (8.9)	0.902
7	139 (47.3)	45 (44.1)	94 (49.0)	
8	126 (42.9)	45 (44.1)	81 (42.2)	
Type of school				
Public	201 (68.4)	63 (61.8)	138 (71.9)	0.100
Private	93 (31.6)	39 (38.2)	54 (28.1)	
Parent’s sociodemographic				
Father’s education level				
Low	202 (68.7)	64 (62.7)	138 (71.9)	0.140
High	92 (31.3)	38 (37.3)	54 (28.1)	
Mother’s education level				
Low	213 (72.4)	69 (67.6)	144 (75.0)	0.228
High	81 (27.6)	33 (32.4	48 (25.0)	
Father’s occupation				
Informal	142 (48.3)	46 (45.1)	96 (50.0)	0.498
Formal	152 (51.7)	56 (38.8)	96 (50.0)	
Mother’s occupation				
Informal	239 (81.3)	75 (73.5)	164 (85.4)	0.020 *
Formal	55 (18.7)	27 (26.5)	28 (14.6)	
Family-related factors				
First childbirth order				
Yes	170 (57.8)	58 (56.9)	112 (58.3)	0.905
No	124 (42.2)	44 (43.1)	80 (41.7)	
Presence of siblings				
No	25 (8.5)	10 (9.8)	15 (7.8))	0.717
Yes	269 (91.5)	92 (90.2)	177 (92.2)	
Parenting style				
Father				
Permissive	7 (2.4)	1 (1.0)	6 (3.1)	0.395
Authoritarian	1 (0.3)	1 (1.0)	0 (0.0)	
Authoritative	286 (97.3)	100 (98.0)	186 (96.9)	
Mother				
Permissive	60 (20.4)	20 (19.6)	40 (20.8)	0.764
Authoritarian	1 (0.3)	0 (0.0)	1 (0.5)	
Authoritative	233 (79.3)	82 (80.4)	151 (78.6)	
Level of dental anxiety				
Father				
High	23 (7.8)	17 (16.7)	6 (3.1)	0.001 *
Low	271 (92.2)	85 (83.3)	186 (96.9)	
Mother				
High	36 (12.2)	21 (20.6)	15 (7.8)	0.001 *
Low	258 (87.8)	81 (79.4)	177 (92.2)	

* Chi-square test; *p*-value < 0.05.

**Table 2:** Logistic regression of several factors associated with dental fear and anxiety among children (*n* = 294).

Variables	Crude OR (95% CI)	*p*-Value	AOR (95% CI)	*p*-Value
Mother’s occupation [reference = informal]	2.10 (1.16–3.82)	0.020	2.01 (1.05–3.85)	0.034
Father’s dental anxiety [reference = low]	6.20 (2.36–16.28)	0.001	4.68 (1.64–13.33)	0.004
Mother’s dental anxiety [reference = low]	3.06 (1.50–6.24)	0.003	2.50 (1.10–5.74)	0.029

**Table 3:** Children’s dental fear and anxiety (*n* = 294).

	Response *n* (%)				
	Not Afraid	Very Little Fear	Moderate Fear	Pretty Much Afraid	Very Much Afraid
Possible triggers					
Dentist	150 (51)	84 (28.6)	44 (15)	11 (3.7)	5 (1.7)
Doctors	205 (69.7)	55 (18.7)	28 (9.5)	3 (1)	3 (1)
Injections	143 (48.6)	71 (24.1)	52 (17.7)	15 (5.1)	13 (4.4)
Somebody examines his/her mouth	232 (78.9)	35 (11.9)	25 (8.9)	0	2 (0.7)
Having to open the mouth	256 (87.1)	20 (6.8)	16 (5.4)	1 (0.3)	1 (0.3)
Having a stranger touch you	58 (19.7)	67 (22.8)	123 (41.8)	19 (6.5)	27 (9.2)
Having somebody look at him/her	88 (29.9)	77 (26.2)	108 (36.7)	14 (4.8)	7 (2.4)
Dentist drilling	48 (16.3)	61 (20.7)	117 (39.8)	29 (9.9)	39 (13.3)
Sight of dentist drilling	53 (18)	63 (21.4)	116 (39.5)	31 (10.5)	31 (10.5)
Hearing drilling sound	79 (26.9)	73 (24.8)	109 (37.1)	18 (6.1)	15 (5.1)
Putting instruments in his/her mouth	100 (34)	78 (26.5)	87 (29.6)	17 (5.8)	12 (4.1)
Choking	50 (17)	57 (19.4)	140 (6.5)	19 (6.5)	28 (9.5)
Having to go to the hospital	198 (67.3)	52 (17.7)	35 (11.9)	5 (1.7)	4 (1.4)
People in white uniform	238 (81)	30 (10.2)	20 (6.8)	2 (0.7)	4 (1.4)
Having the dentist clean his/her teeth	201 (68.4)	66 (22.4)	22 (7.5)	2 (0.7)	3 (1)
Median of CFSS-DS score (min–max)	28 (15–75)				

## Data Availability

The data presented in this study are not publicly available due to privacy and ethical restrictions, as the dataset contains individual-level information from child–parent participants. The data may be made available from the corresponding author upon reasonable request and subject to approval by the relevant ethics committee.
